# Differential Contribution of the Repeats to Heparin Binding of HBHA, a Major Adhesin of *Mycobacterium tuberculosis*

**DOI:** 10.1371/journal.pone.0032421

**Published:** 2012-03-05

**Authors:** Pierre Lebrun, Dominique Raze, Bernd Fritzinger, Jean-Michel Wieruszeski, Franck Biet, Alexander Dose, Mathieu Carpentier, Dirk Schwarzer, Fabrice Allain, Guy Lippens, Camille Locht

**Affiliations:** 1 INSERM U1019, Lille, France; 2 CNRS UMR 8204, Lille, France; 3 Institut Pasteur de Lille, Center for Infection and Immunity of Lille, Lille, France; 4 Univ Lille Nord de France, Lille, France; 5 CNRS UMR8576 Structural and Functional Glycobiology, Villeneuve d'Ascq, France; 6 Interfaculty Institute for Biochemistry, University of Tübingen, Tübingen, Germany; 7 UR1282, Infectiologie Animale, Sante' Publique (IASP-311), INRA, Nouzilly, France; Institut de Pharmacologie et de Biologie Structurale, France

## Abstract

**Background:**

Tuberculosis remains one of the most important causes of global mortality and morbidity, and the molecular mechanisms of the pathogenesis are still incompletely understood. Only few virulence factors of the causative agent *Mycobacterium tuberculosis* are known. One of them is the heparin-binding haemagglutinin (HBHA), an important adhesin for epithelial cells and an extrapulmonary dissemination factor. HBHA mediates mycobacterial adherence to epithelial cells via the interactions of its C-terminal, lysine rich repeat domain with sulfated glycoconjugates on the surface of epithelial cells.

**Methodology/Principal Findings:**

Using defined heparin sulfate (HS) analogs, we determined the minimal heparin fragment length for HBHA binding and structural adaptations of the HBHA heparin-binding domain (HBD) upon binding to heparin. The NMR studies show significant shifts of all residues in the HBD upon interaction with heparin, with stronger shifts in the last repeats compared to the upstream repeats, and indicated that the HS fragments with 14 sugar units cover the entire C-terminal lysine-rich domain of HBHA. The differential implication of the repeats is determined by the relative position of prolines and lysines within each repeat, and may contribute to binding specificity. GAG binding induces a non-homogeneous structural rearrangement in the HBD, with stabilization of a nascent α-helix only in the last penta-repeats.

**Conclusion/Significance:**

Mycobacterial HBHA undergoes structural adaptation upon interaction with GAGs, which is likely involved in binding specificities of the adhesin, and mycobacterial pathogens may use HBD polymorphisms for host or organ specificity. Further studies will aim at decoding the complementarity between HBD repeats and HS sequence.

## Introduction

Tuberculosis is a world-wide leading cause of mortality due to an infectious agent. Because of the complexity of the disease and the virulence of its causative agent *Mycobacterium tuberculosis*, the molecular mechanisms of pathogenesis are still incompletely understood [1]. One of the few characterized mycobacterial virulence factors is the cell wall-associated Heparin-binding Haemagglutinin (HBHA) (for review, see [2]). It was initially identified as one of the major adhesins involved in binding of *M. tuberculosis* to the epithelial cells via interactions with heparan sulfate (HS) Glycosaminoglycans (GAGs) [3], [4], [5]. Subsequently, it was found to be a key factor in the extrapulmonary dissemination of the bacilli [6], [7], [8]. In addition to being an important virulence factor, HBHA is also a potent protective antigen [9], [10] that may be used as diagnostic tool, in particular for the detection of latently infected subjects [11], and as a vaccine candidate, especially to strengthen and prolong Bacille Calmette & Guérin (BCG)-induced immunity [12], [13], [14].

HBHA is a 198 residues protein organized in four domains : a hydrophobic domain (residues 5 to 18) [15], a coiled-coil domain (24 to 109) [15], [16], [17], a linker domain (110–159) [17] and a cationic lysine-rich domain (160 to 198) [5]. The latter domain is essentially composed of alanine, proline and lysine residues, organized in two types of lysine-rich repeats, R1 and R2. R1 (KKAAPA) is directly repeated thrice between residues 160 and 177, whereas R2 (KKAAAKK) is repeated twice between residues 178 and 194, and both R2 repeats are separated by APA. The sequence can also be read as a repetition of three hexa-repeats (KAAPAK) and three penta-repeats (KA(A/P)AK), whereby the two lysine residues of these latter are separated by a triple A or by a APA tripeptide. This latter reading will be adopted in this manuscript. These repeats constitute the heparin-binding domain (HBD) of HBHA and are responsible for binding of HBHA to Heparan Sulfate (HS) GAGs [5].

Complex HS-GAGs are found on cell surfaces and in the extracellular matrix of most animal tissues. Many secreted proteins bind to these polysaccharides, usually to the heparin-like regions of HS. The essential role of protein-GAG interactions in the regulation of various physiological processes has been recognized since several decades, but only recently the molecular bases underlying these interactions have emerged [18]. The cell surface GAGs are actively involved in barrier regulation; interactions with cationic peptides induce signaling events that lead to increased endothelial permeability and cytoskeletal reorganization [19]. Previous work has shown that the repeats mediate the binding of HBHA to respiratory epithelial cells [5], and that treatment of these same epithelial cells with heparinase III leads to a strong reduction in HBHA binding. Furthermore, anti-HBHA monoclonal antibodies were shown to effectively disrupt the interaction between HBHA and epithelial cells, thereby reducing the extrapulmonary dissemination of the bacteria [20]. HBHA-coated beads are able to transcytose epithelial cell layers and induce cytoskeletal rearrangements [8]. The interactions of HBDs with HS are dominated by electrostatic forces between basic amino acid residues, such as lysines, and the negatively charged sulfates of HS. Therefore, heparin is commonly used as a model compound to study these interactions.

Heparin is a repeating linear copolymer of α-L-uronic acid or β-D-glucuronic acid (1→4)-linked to a glucosamine residue. Whereas the most common structure in heparin is the trisulfated disaccharide, a number of structural variants exist, rendering it micro-heterogeneous. Heparin is polydisperse with an average chain length of 13 kDa in commercial heparin, and ranges from 5 to 40 kDa. Initial structure-function studies of HBHA have used micro-heterogeneous and polydispersed commercial heparin. To gain further insights into the HBHA-HS interactions, we use here size-defined heparin-derived oligosaccharides to determinate the minimal length required for binding to HBHA. With these defined oligosaccharides, we then set out to characterize the resulting complex at the molecular level.

## Results

### Defining the minimal binding partners

**Figure 1:**
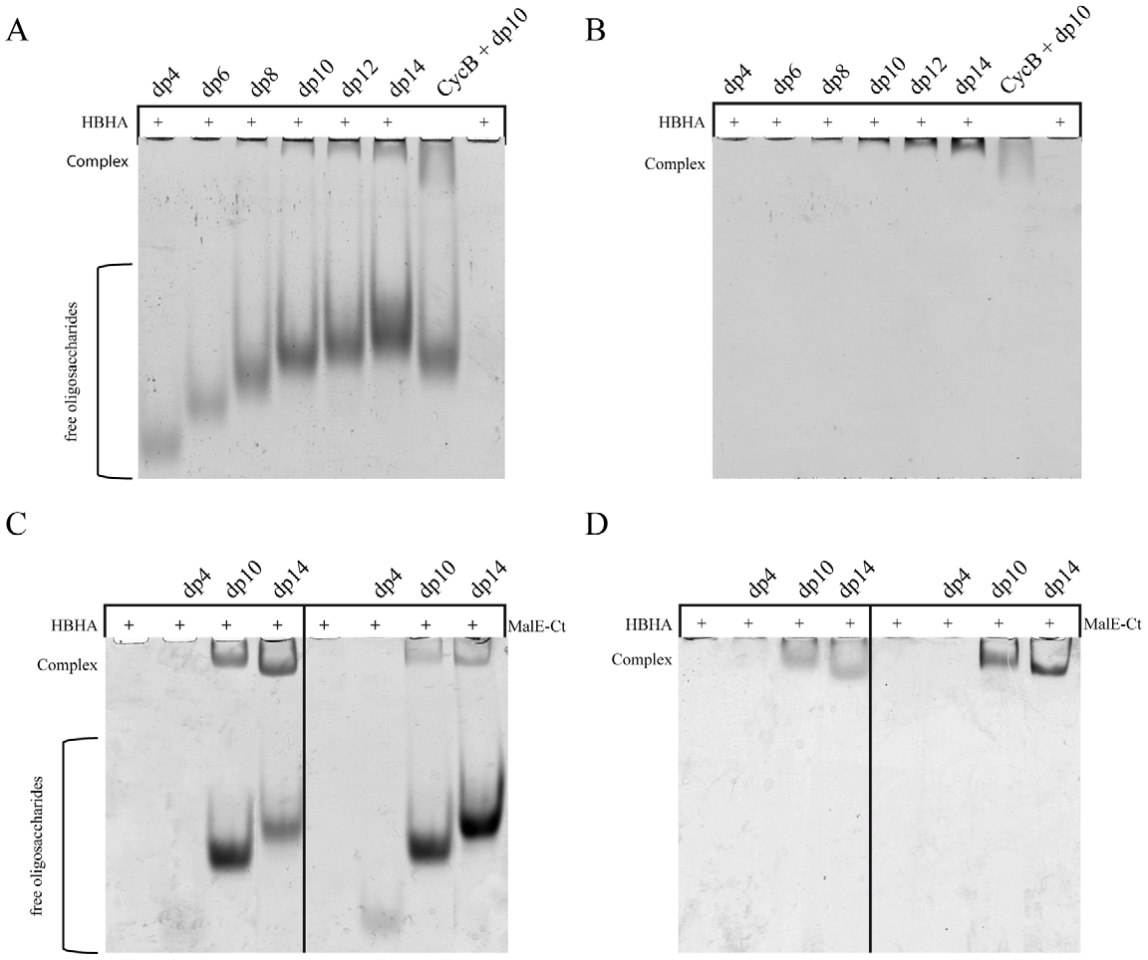
Analysis of the oligosaccharide minimal unit length required for HBHA binding. Oligosaccharides from dp4 to dp14 (5 nmol of each dp oligosaccharide per lane) were incubated with 0.2 nmol of HBHA (A & B) or MalE-Ct (C & D) and subjected to mobility shift assays. The migration of the oligosaccharides was visualized by coloration with azur blue (A & C) and that of the proteins by Coomassie blue staining (B & D). Cyclophilin B (CycB) and free HBHA or MalE-Ct were used in the positive and negative control lanes, respectively.

To determine the minimal length of the heparin fragment required for HBHA binding, heparin-derived oligosaccharides ranging in size from dp4 to dp14 were used in a mobility shift assay. The oligosaccharides (5 nmol per sample) were incubated with 0.2 nmol of HBHA and subjected to non-denaturant electrophoresis (Figure 1A and B). In these conditions, HBHA is positively charged and cannot shift to the anode. However, charge compensation by complex formation with negatively charged heparin fragments allows the migration of the complex, and can thereby be visualized as a shifted band at the top of the gel. The unbound oligosaccharides in excess are visible in the middle region of the gel. Tetra- and hexa-saccharides were not significantly shifted in the presence of HBHA, indicating that they were inefficient in forming complexes with HBHA. In contrast, significant amounts of octasaccharides and larger oligosaccharides were shifted in the presence of HBHA. Cyclophilin B (CycB) and dp10 were used as positive controls for interaction, as the heparin:CycB interaction was previously described and HBHA and Cyclophilin B have similar values for pI and molecular weight [21]. These results indicate that the minimal length of heparin capable of interacting with HBHA is between 8 and 10 sugar units. For subsequent studies, we used the dp14 fragment.

The chimeric protein consisting of the maltose-binding protein (MBP) and the HBD domain of HBHA grafted onto its C-terminus (MalE-Ct) showed the same affinity as HBHA for heparin as measured by surface plasmon resonance, and hence allowed to ascertain that the tail region of HBHA is solely responsible for HS binding [5]. We here find that it exhibits the same profile on the gel mobility shift assays in the presence of our heparin derivatives (Figure 1, C and D). The C-terminal domain hence recognizes the same heparin length distribution when integrated into full-length HBHA or when grafted onto the C-terminus of MBP.

### Structural independence of the HBD

**Figure 2:**
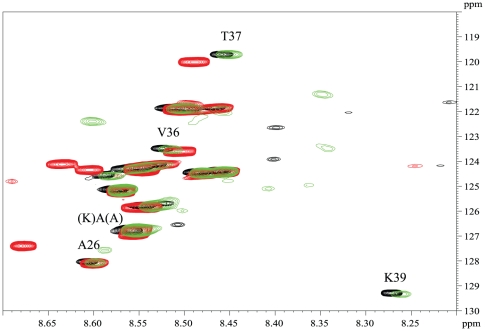
^1^H, ^15^N HSQC at 4°C and 900 MHz spectrum of recombinant U-^15^N labeled HBHA (green), U-^13^C, ^15^N labeled MalE-Ct (black) and the synthetic peptide at natural abundance (red). Sequence-specific assignments are indicated.

To further ascertain that the HBD is independent of the rest of the protein, we recorded ^1^H, ^15^N HSQC spectra of ^15^N-labeled samples of both proteins (Figure 2). Although more resonances were visible in the spectrum of HBHA, possibly reflecting the presence of the flexible linker region that was shown to stabilize the coiled coil region of HBHA [17], the only resonances of MalE-Ct that were visible perfectly match a subset of the HBHA resonances (Figure 2). Finally, we recorded under similar conditions the ^1^H, ^15^N HSQC spectrum at natural abundance of a synthetic peptide corresponding to the HBD. We recovered the peaks common to HBHA and MalE-Ct for most residues of the synthetic peptide. Only for the extreme N- and C-terminal positions we observed slightly different chemical shift values, which can be related to the lack of any N-terminal extensions (the rest of the HBHA protein or Mal) ) and C-terminal amidation of the synthetic peptide (Figure 2).

### Molecular characterization of HS binding by NMR assignment of MalE-Ct bound to dp14

Resolving individual resonances of the HBD was attempted by triple resonance NMR spectroscopy on a ^13^C, ^15^N-labelled MalE-Ct sample. Despite the high magnetic field corresponding to a proton frequency of 900 MHz, the majority of resonances could not be resolved or assigned unambiguously in the highly repetitive sequence. Only some residues of the C-terminal part and of the non-degenerate 5^th^ repeat sequence _183_KKAPA_187_ could be assigned in a sequence-specific manner (Figure 2). For the other resonances, the nature of the corresponding amino acid (and its (i-1) neighbour) could be deduced from the carbon Cα and Cβ chemical shift values, but a sequence-specific assignment was not possible.

**Figure 3:**
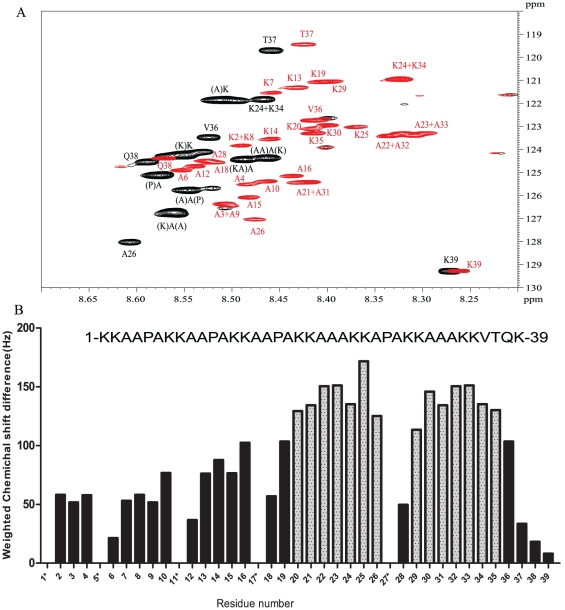
(A) HSQC spectrum of U-^13^C, ^15^N labeled MalE-Ct at 4°C and 900 MHz (black) and of the same sample after addition of a 5.6-fold excess of dp14 (red). Sequence-specific assignments of the HBD are indicated. (B) Plot of weighted chemical shift differences in 2D ^1^H/^15^N HSQC spectra of HBD-bound vs free HBD as a function of residue number. Residues with the most significant chemical shift changes upon dp14 binding are shown in grey. Proline residues and residues that could not be assigned are marked with an asterisk.

Next, we recorded the HSQC spectrum of MalE-Ct in the presence of a fivefold excess of dp14, and observed a general upfield shift of most resonances, with shifts attaining 0.15 ppm in the proton direction (Figure 3A). In addition to the shifts, the number of peaks in the spectrum greatly increased, suggesting that the interaction with dp14 reduces to some extent the degeneracy of the different repeats. An example is given by the intense resonance at 8.56/126.73 ppm, that we assigned to the (K)A(A) motif in the spectrum of MalE-Ct without dp14 (Figure 3A), and that corresponds to the amide correlation of 5 Ala residues in the HBD. Upon interaction with dp14, this resonance splits up into five individual resonances that could be divided in two groups. The first group contains three distinct peaks, that we assigned to the (K)A(AP) resonance in the first three repeats (A3, A9 & A15, see below), whereas the second group contains the (K)A(AA) motif of the fourth and sixth repeat (A21+A31 see below). Remarkably, the shift of the resonances in the second group is almost twice that of the resonances in the first group.

The Cα/Cβ carbon chemical shift values unambiguously indicate the nature of the amino acid and its (i-1) upstream neighbour, but do not allow us to connect individual resonances, as the degeneracy at the level of the carbon chemical shift values remains even in the presence of a saturating amount of dp14. As an example, the Cα/Cβ carbons of the central Ala residue of the previously mentioned (K)A(A) motif. adopt values of 52.2/19.2 ppm in the first three repeats, close to the values of 52.6/19.02 ppm predicted on the basis of a set of intrinsically unstructured proteins (Table S1: predicted chemical shifts). In repeats 4 and 6, where an Ala rather than a Pro residue follows the KAA tripeptide, the values are 52.6/19.04 ppm, which is even closer to these predicted random coil values. The carbonyl CO resonances show the same degeneracy. Therefore, only the reasonable ^15^N dispersion could be exploited in the third indirect dimension of the HNN experiments to link peaks together. As a result, peaks could be assembled corresponding to every single repeat (Figure S1). Not unexpectedly, resonances in the first three hexa-repeats (defined here as KAAPAK) can be grouped by their weak, intermediate or strong shift.

In order to unambiguously assign the resonances to the individual repeats, we synthesized a peptide with a single ^15^N-Ala in every repeat. As expected, we obtained a spectrum with only 6 resonances that coincide perfectly with the resonances of the corresponding Ala residues in U-^15^N labelled MalE-Ct, (Figure S2). Upon addition of a tenfold excess of dp14 to the peptide, while maintaining the same absolute concentration of 60 µM peptide, cross peaks shifted in a manner similar to those observed with the recombinant proteins. Superposition of the peptide spectra allowed us to establish that shifts of the resonances in the first group corresponding to the first three repeats obey the order of occurrence, with the smaller shifts observed for residues in the first group, intermediate shifts in the second repeat and the most pronounced shifts in the third repeat. A full sequence-specific assignment of this highly degenerate C-terminal peptide was thus possible in the presence of dp14 (Figure 3), and allowed us to conform that shifts in the 4^th^ and 6^th^ KAAAK penta-repeats are more pronounced (Figure 3B).

### GAG binding induces structural changes

**Figure 4:**
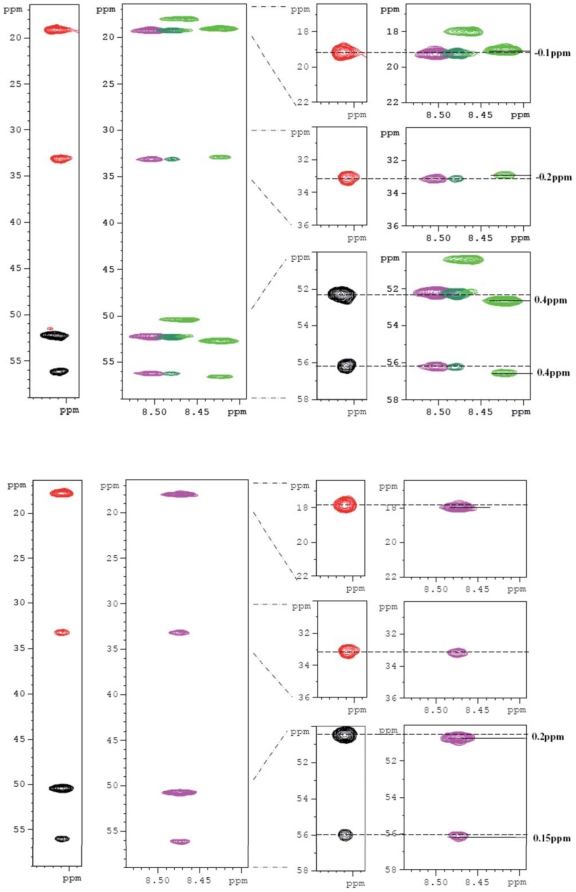
Strips through the HNCACB peaks of isolated MalE-Ct (black and red, for positive and negative contours), and of the MalE-Ct with an excess of heparin (colours). The top panel shows the degenerate (K)A(A) resonance (left panel, black and red; and corresponding zooms) in isolated HBHA, that splits in the (K)A(AP) resonances (pink and dark green) and in the (K)A(AA) resonances of R4 and R6 (light green). The bottom panel shows the strips through the isolated (K)A(PA) resonances of R5.

In order to evaluate potential structural changes upon heparin binding, we looked for Cα and Cβ chemical shift differences upon dp14 binding. The degenerate (K)A(A) amide cross peak equally shows that in isolated HBHA, the Cα and Cβ of both the first K and second A residue are identical for all repeats, R5 excepted (Fig. 4, left panel, black and red; and corresponding zooms). After splitting into (K)A(AP) resonances of R1–R3 and (K)A(AA) resonances of R4 and R6 (Figure 3B), we find that the Cα and Cβ resonances of the K and A residues in the latter R4 and R6 repeats shift towards more α-helical values (Figure 4, top, light green). The Cα shift of 0.4 ppm would not classify those residues as a stable helix according to the Chemical Shift Index [22], but does indicate a nascent α-helix [23], [24], [25]. The equivalent residues in the R1–R3 (K)A(AP) repeats show no or slightly opposite variations (Figure 4, top, pink and dark green), showing that they cannot evolve towards a more helical conformation upon heparin binding. When we apply the same analysis for the unique (K)APA(K) R5 repeat, chemical shift differences are smaller but evolve in the same direction as the KAAAK R4–R6 pentarepeats This unexpected result indicates that a central Pro in the repeat still is compatible with a helix induction through heparin binding, whereas the same Pro integrated in a hexarepeat imposes a more extended conformation to accommodate the heparin binding.

### Thermodynamic characterization of the interaction of HBHA with dp14

The differential shifts for similar residues in different repeats could originate from several independent binding events or from the same binding event that is perceived in a differential manner by the individual repeats. To distinguish between these two possibilities, we determined the apparent dissociation constant by recording the ^1^H, ^15^N HSQC of MalE-Ct with increasing amounts of dp14 in a NMR titration experiment (Figure S3B). The observed gradual peak shifts clearly indicate that we are in the fast exchange regime on the NMR time scale, allowing to extract dissociation constants by fitting the corresponding chemical shift variations to the dp14 concentrations. A typical titration curve is shown in Figure S3F, and indicates a K_D_ value of 58.8±14 µM for dp14 (Figure S3D). When this value was extracted on different resonances, the same range of values was found for all of them (Figure S3D and E), suggesting that we observed the binding of a single dp14 to the HBD of HBHA (Figure S3). We performed the same titration experiment with the shorter dp10 and determined a K_D_ of 79.4±23 µM (Figure S3D and F) indicating a slightly less tight interaction than for dp14, in agreement with our earlier gel shift results (Figure 1). Again, the values were identical irrespective of the resonance used. However, when dp4 was added to the same protein, hardly any shift was observed (Figure S3C), which is in agreement with the lack of binding of dp4, as evidenced by the gel shift assays (Figure 1).

### A penta- and hepta-repeat code for different mycobacterial species?

**Figure 5:**
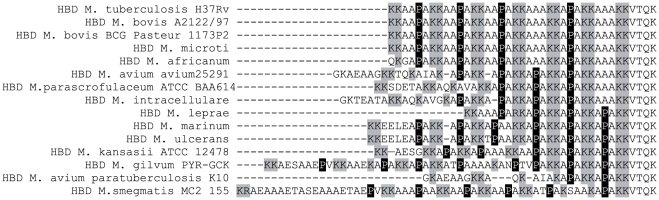
Multiple alignment of HBHA HBDs from different *Mycobacterium* species. Alignments were done using the Kalign algorithms [41]. Conserved lysine residues are highlighted in grey and conserved proline residues in black.

Chemical shift differences upon complex formation with dp14 are more pronounced for the penta-repeats 4, 5 and 6 than for the three N-terminal hexa-repeats, and are moreover accompanied by Cα and Cβ changes that indicate a more pronounced helical tendency upon heparin binding. The pronounced shifts observed for residues in penta-repeat 5, characterized by a central proline residue (KAPAK), might result from its strong contribution to the binding, but, alternatively, it might also stem from the presence of the two flanking KAAAK penta-repeats 4 and 6 that themselves enhance binding. We therefore examined the distribution of equivalent repeats in different mycobacterial species (Figure 5). Whereas the C-terminal domains of HBHA from at least 4 species align perfectly with that of *M. tuberculosis* HBHA, *Mycobacterium avium* subsp. *avium* (*Maa*) has a HBHA sequence that contains mostly penta-repeats with a central proline residue. Only the last repeat is of the KAAAK type.

**Figure 6:**
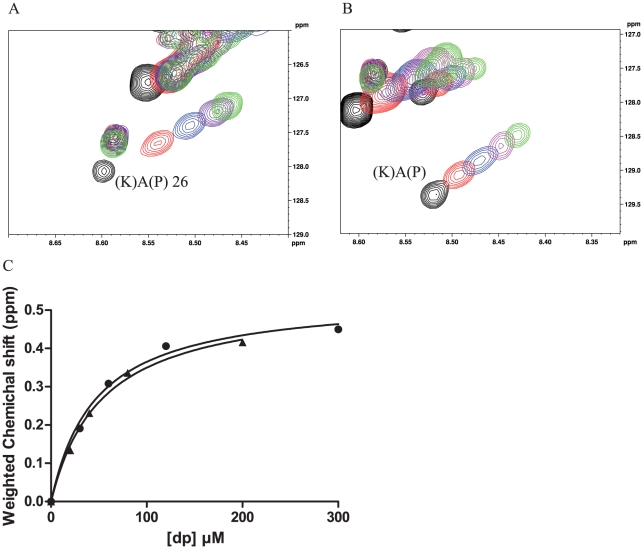
Determination of dissociation constants by NMR titration. Representative ^1^H, ^15^N HSQC NMR spectra of *M. tuberculosis* HBHA (A) and *Maa* HBHA (B) with increasing amounts of dp14 (free HBHA (black), 0.5 dp14:HBHA (red), 1 (blue), 2 (purple), 5 (green)), whereby the peak shifts were used for the determination of the dissociation constants. Chemical shift differences were calculated as 
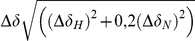
 with Δδ_H_ and Δδ_N_ the observed chemical shift changes for ^1^H and ^15^N, respectively. For the determination of dissociation constants, Δδ was plotted as a function of the molar ratio (dp14:protein) The derived titration curves are shown in (C) for the *M. tuberculosis* HBHA (•) and the *Maa* HBHA (▴).

In order to investigate whether this sequence divergence influences the heparin-binding properties, we compared the ionic strength required to elute recombinant HBHA of *M. tuberculosis* and *Maa* from a heparin sepharose column. Both proteins eluted at 0.59 M NaCl, suggesting that their binding to unfractionated heparin is comparable. Next, we produced *Maa* HBHA in *Escherichia coli* as a ^15^N-labelled protein, and performed comparative titration experiments with dp14. Similar shift differences were observed for *Maa* HBHA and for HBHA from *M. tuberculosis* (Figure 6). The derived dissociation constants for *Maa* HBHA (K_D_ = 47 µM±8) closely match those determined for *M. tuberculosis* HBHA (K_D_ = 53±6 µM) (Figure S3D). These observations suggest that the presence of penta-repeats defines the affinity towards HS, irrespectively of the presence of a central proline. Interestingly, the HBHA sequence of *Mycobacterium smegmatis* contains only a single C-terminal penta-repeat, with a central proline residue (Figure 5). Previous studies have shown that the ionic strength required to elute *M. smegmatis* HBHA from heparin-sepharose is significantly lower than for the *M. tuberculosis* HBHA [26], and that the *M. smegmatis* HBHA does not bind epithelial cell surfaces.

## Discussion

The interaction between GAGs and the HBD of HBHA is one of the key events in the biological activities of HBHA, as it is the basis of adherence of *M. tuberculosis* to epithelial cells [5] and of the subsequent extrapulmonary dissemination of the bacilli. HBHA-like molecules are produced by all mycobacteria investigated so far [2], and are likely to play important roles in the virulence of pathogenic mycobacteria, such as *M. avium* spp [27] and *Mycobacterium leprae* [*28*], but their role in non-pathogenic mycobacteria is less clear [26]. We report here the molecular and structural characterization of this interaction. By using heparin-derived oligosaccharides of defined lengths we defined the minimal size of oligosaccharide for the interaction between HBHA and the HS. In other models the use of defined oligosaccharides has been validated as an appropriate tools to study the molecular interactions of HBD with HS chains [29], and many structural and biological studies using defined oligosaccharides were consistent with the *in vivo* biological data [30]. We observed that the minimal binding unit for HBHA is a dp8/dp10. This was determined by gel mobility shift assays and confirmed by NMR titration experiments. This is in apparent contradiction with our earlier report of a 3 kDa heparin fraction not protecting the recombinant HBHA against protéolytic degradation [5]. However, analysis of the gel filtration profile of the previously used fractions showed that they contained a major fraction of very short dp2/dp4 oligosaccharides, that indeed interact very poorly with HBHA (Figure 1). The heparin fragments of this study were purified and controlled by HPLC, are therefore more homogeneous in size and allow to set the lower limit for HBHA interaction at the level of a dp8.

By using NMR titration experiments, the dp14 K_D_ value for the interaction with HBHA was determined to be 53±6 µM. This value is consistent with the ionic strength of 0.59 M NaCl necessary to elute HBHA from a heparin-sepharose column [5]. K_D_ values in the µM ranges have been associated with elution from heparin-sepharose at concentrations of 0.3–0.4 M NaCl [31].

The main conclusion of our NMR titration experiments is that we can deconvolute in detail the contribution of the different repeats within the HBD of HBHA. Because the interaction of the HBD with a saturating amount of dp14 lifts, to some extent, the degeneracy of the NMR spectrum, we could assign all the individual cross peaks in the HSQC spectrum (Figure 3). As a result of this per-residue view of the molecular interaction, we can come to the following two conclusions. First, the K_D_ values derived from the shift of cross peaks for the same residue in each repeat are similar. This argues against the existence of more than one binding site with differential affinity. Secondly, the more pronounced chemical shift differences residues in the R4–R6 penta-repeats suggests that the lysines in the C-terminal penta-repeats are more directly involved in the molecular interaction of the HBHA HBD with dp14. Carbon chemical shift values give an explanation for this, as they indicate an increased helical tendency only for residues in these R4–R6 repeats. Previous studies with model peptides composed of only Lys and Ala residues indicated that an alternating spacing of 3 and 4 Ala residues between 2 Lys residues was optimal for presenting the lysine side chains to the negative sulfate clusters on the heparin surface [32], thereby strongly stabilizing a heparin-induced α-helical structure of the model peptide [32]. The HBHA sequence of *M. tuberculosis* deviates in two aspects from this model peptide. First, instead of individual lysine residues, as in the model peptide, the HBHA HBD contains di-lysines sequences (Figure 5). Secondly, the residues that separate two consecutive lysine clusters are canonical Ala triplets only in repeats 4 and 6, whereas they contain a central proline residue in all other repeats. However, the HS K_D_ value of 80 µM observed for the interaction with the Lys-Ala model peptide (35) is very similar to that of dp14 found here for the interaction with the HBHA HBD. In general, heparin-binding proteins feature a large range of HS affinities, with the determining factor being the spatial display of the positive charges. Our results indicate that the repeats in HBHA can adapt differentially to the presence of HS to provide charge and/or structure complementarity.

Only few examples of GAG-binding domains with prolines have been described. Using a synthetic 7-mer random peptide library composed of the 20 natural amino acids, Caldwell *et al.* [33] have found proline-containing peptides after purification on GAG-sepharose. It has been suggested that the reverse turn induced by proline is important for the interaction of hepatocyte growth factor with heparin [34]. Furthermore, in the vaccinia virus envelope protein A27, a turn-like structure introduced by a single KKPE segment has been shown to be responsible for its specific binding to HS [35]. Here, we describe a novel example, in which the insertion of a proline residue between two lysines allows to maintain a strong affinity of the mycobacterial HBHA HBD for GAGs. The precise reason for the proline insertion within the lysine-rich repeats remains to be determined.

The observation that cross peaks belonging to residues of the proline-containing hexa-repeats shift significantly less than their C-terminal counterparts led us to consider the HBHA primary sequences of the different mycobacterial species as a code for heparin binding. The *Maa* HBHA contains only a single KAAAK penta-repeat, but contains four consecutive KAPAK penta-repeats. Despite this, the affinity of this HBD for dp14 was similar as that of the *M. tuberculosis* HBD, as evidenced by NMR titration experiments. Therefore, we conclude that a proline in a penta-repeat is more compatible with HS binding than the same proline in a hexarepeat. Considering the variability of the cellular HS-GAGs, it is _ENREF_29tempting to speculate that mycobacterial pathogens use HBD variability for host or organ specificity [36]. Further investigation of this complemantrity and subsequent specificity is currently ongoing in our laboratory.

## Materials and Methods

### Bacterial strains, growth conditions and DNA manipulations

*Maa* ATCC 25291 was grown at 37°C in Sauton medium [37] or Middlebrook 7H9 broth (Difco Laboratories, Detroit, MI), with 0.2% glycerol and albumin-dextrose-catalase (ADC) enrichment medium (Becton Dickinson, Le Pont de Claix, France). Bacteria were harvested at mid-log phase and kept frozen (−80°C) in aliquots until further use. *E. coli* BL21(DE3) (Novagen, Darmstadt, DE), *E. coli* TOP10 (Invitrogen Carsbad, CA) and *E. coli* XL1-Blue (Stratagene, Le Jolla, CA) were grown in LB medium [38] supplemented with 30 µg/mL kanamycin as appropriate. Restriction enzymes, T4 DNA ligase and other molecular biology reagents were purchased from New England Biolabs, Roche or Promega. PCRs were performed using a Bio-Rad thermal cycler model iCycler, and the PCR products were sequenced by GenomExpress (Grenoble, France).

### Cloning, sequencing of the Maa HBHA-coding gene

The HBHA-coding gene (Genbank accession number: JN129485 ) was amplified by PCR from chromosomal DNA of strain *Maa* ATCC 25291 using the *Pfu* DNA polymerase (Promega) and two synthetic oligonucleotides (Sigma) with the following sequences: 5′- TATACATATGACCATGGCGGAAAACCCGAACATCG -3′ (*Maa*-hbha S) and 5′- ATATAAGCTTGGTACCCACGAGGTGGTTCACGCC -3′ (*Maa*-hbha AS), containing a *Nco*I and a *Hin*dIII site, respectively (underlined). The fragment was amplified after a short denaturation cycle of 3 min at 95°C by using 35 cycles as follows: 95°C for 30 s, 57°C for 30 s, and 72°C for 30 s with a final elongation cycle at 72°C for 10 min. The PCR product was inserted into pCR2.1-TOPO (Invitrogen) after the addition of an adenylated extension according to the instructions of the supplier. After sequencing, the fragment containing the HBHA-coding sequence was digested by *Nco*I and *Hin*dIII and then inserted into pET-24d(+) (Novagen), generating pET::Maahbha. This plasmid was used to transform *E. coli* XL1-Blue for sequencing.

### Production of recombinant HBHA derivatives

Recombinant full-length HBHA from BCG and *Maa* 25291 and the maltose-binding protein (MBP) containing the C-terminal, lysine-rich domain of HBHA (MalE-Ct) were produced in *E. coli* BL21(DE3) and B834, respectively. Electrocompetent BL21(DE3) and B834 were transformed with pET-HBHA and pMAL+3R1+2R2 [5], respectively, and transformants were selected on LB agar plates containing respectively 25 µg/mL kanamycin and 50 µg/mL ampicillin. From a single colony, an overnight culture (20 mL) was grown at 37°C in LB media supplemented with the relevant antibiotics. These overnight cultures were used to inoculate 2 L of the same media at a 1/100 dilution for the production of recombinant proteins used in gel shift assays and in 2 L of M9 ^15^N or ^15^N/^13^C+0.1% Isogro ^15^N or ^15^N/^13^C (Sigma-Aldrich, France) for the production of recombinant proteins used in NMR studies. We did note that production in the minimal medium led to less protein degradation than in the rich LB medium. The resulting cultures were grown to an OD600 value of 0.8 and then treated with 1 mM isopropyl-dithiogalactoside and grown for an additional 4 h at 37°C. Cells were harvested by centrifugation at 5,000× *g* for 20 min at 4°C and stored frozen at −20°C until further use.

### Protein purification

Frozen cell pellets were resuspended in 40 mL 20 mM phosphate-buffer containing 0.15 mM NaCl, 0.1% Triton X100, supplemented with COMPLETE free inhibitor cocktail EDTA-free (Roche) and DNaseI (10 µg/mL). The cells were disrupted using a French press cell, and lysates were clarified by centrifugation at 17,000× *g* for 30 min at 4°C. The bacterial cell extracts were then loaded onto a 1 mL Heparin Hi-trap column (GE Healthcare) equilibrated in 20 mM phosphate buffer containing 0.15 M NaCl, washed with 20 mM phosphate buffer containing 0.25 M NaCl and eluted with 20 mM phosphate buffer containing 0.65 M NaCl. The HBHA- or MalE-Ct-containing fractions were pooled and then fractionated by size exclusion chromatography using a Superdex 200 prepgrade (GE Healthcare) in 20 mM phosphate buffer containing 0.15 M NaCl (pH 7.4) for gel shift assays or 20 mM phosphate buffer containing 0.15 M NaCl (pH 6.8) for NMR. Finally, the protein samples were concentrated by VIVASPIN 20 (10 000 MWCO PES) at 4°C.

### Preparation of heparin-derived oligosaccharides

Sixty-two mg of low-weight porcine heparin was fractionated by filtration on Bio-Gel P-6 (Bio-Rad laboratories, Hercules, CA) in NH_4_HCO_3_ as described [21]. For NMR, commercial heparin-derived oligosaccharides (tetrasaccharides : dp4, decasaccharides: dp10 and dp14) were used (DEXTRA, UK). NMR and HPLC analysis of these latter oligosaccharides showed that they were homogeneous in size and composition. 75% of heparin is IdoA,2S - GlcNS,6S.

### Gel mobility shift assay

HBHA and oligosaccharides were mixed in 40 µL of binding buffer (10 mM Tris-HCl, pH 7.5, 200 mM NaCl, 50 mM KCl, 1 mM EDTA and 0.5 mM DTT) for 30 min at 20°C [21]. The samples were then supplemented with 10 µL of 60% glycerol and subjected to electrophoresis in a 10% (w/v) native polyacrylamide gel in 10 mM Tris, 1 mM EDTA (pH 7.4). Electrophoresis was carried out at 100 V for 30 min. Bromophenol Blue was used as electrophoresis markers. At the end of the electrophoresis, oligosaccharides were stained by Azur blue. The gel was de-stained in water and stained by Coomassie blue to visualize the proteins.

### Peptide synthesis

Amino acid (AA) derivatives were obtained from GLS (Shanghai, China). Coupling reagents were purchased from Merck Novabiochem (Darmstadt, Germany), Tentagel RAM resin from Rapp Polymere (Tübingen, Germany). *N*-(9-Fluorenylmethoxycarbonyl)-L-alanine-^15^N and all other chemicals were purchased from Sigma-Aldrich (Steinheim, Germany). Analytical RP-HPLC was performed on a Varian ProStar 210 HPLC system and on a Shimadzu 10A HPLC system, equipped with a Nucleosil C18 column (5 µm, 4,6×250 mm, Machery-Nagel), employing 0.1% TFA in water (A) and 80% ACN, 0.1% TFA in water (B), as eluents. The analytical gradient was 5–95% B over 50 min with a flow rate of 1 ml/min. Preparative purifications were performed on a Varian ProStar 210 HPLC system equipped with a preparative Dynamax C18 column (10 µm, 21,4×250 mm, Varian) and a flow rate of 13 mL/min. The peptides were analyzed by MALDI-MS on a MALDI-TOF-TOF, 4700 Proteomics Analyzer (Applied Biosystems) and by analytical RP-HPLC.

The peptide was synthesized using standard Fmoc-based solid-phase chemistry on an Intavis Respep XL synthesizer in a 25 µmol scale. TentaGel R RAM resin (loading 0.19 mmol/g) served as solid support and amino acid side-chains were protected as follows: Lys(Boc), Gln(Trt) and Thr(tBu). Coupling reactions were achieved by using 2-(1H-benzotriazole-1-yl)-1,1,3,3-tetramethyluronium-hexafluorophosphate (HBTU) as the activation agent and N-Methylmorpholine (NMM) in DMF/NMP as base. Each successive amino acid was doubly coupled in 5-fold molar excess. Removal of the Fmoc group was carried out with 20% piperidine in DMF.

The ^15^N-labeled alanine residues were introduced manually in a single coupling reaction using 2-(7-aza-1H-benzotriazole-1-yl)-1,1,3,3-tetramethyluronium hexafluorophosphate (HATU) and NMM in DMF. These reactions were performed in 4 molar excess for 1 h with a mixture of labeled Fmoc-amino acid (100 µmol), HATU (90 µmol) and NMM (0.6 mmol) in DMF followed by a capping step using Ac_2_O and N,N-Diisopropylethylamine (DIPEA) in DMF. The peptide was cleaved off the resin with complete removal of the side-chain protection groups with a cleavage cocktail that contained TFA/Phenol/Triisopropylsilane/H_2_O (85∶5∶5∶5) for four hours. The cleaved product was precipitated in cold diethyl ether, centrifuged and washed with diethyl ether, dissolved in H_2_O and lyophilized. The crude peptide was purified with preparative RP-HPLC.

### NMR spectroscopy

NMR samples were prepared in 20 mM phosphate buffer (pH 6.8), 0.15 M NaCl, 1 mM perdeuterated trimethyl silyl propionate (TMSP-*d4*) as a reference and 5% D2O. NMR spectra were recorded at 4°C and 20°C on a Bruker 600 Avance II or Bruker 900 Avance III spectrometer, both equipped with a cryogenically cooled probehead. HSQC spectra were recorded with the standard Bruker sequence for the sensitivity enhanced HSQC with gradient based water suppression, with 16 scans per increment and 4 k×256 points in the t2, t1 directions. Fourier transformation was done after zero filling and multiplication with a π/4 and π/3 shifted square sine bell function for the t2 and t1 directions, respectively. For the assignment, we recorded HNCACB [39] and HN(CA)NNH [40] spectra at 4°C and 900 MHz on a 100 µM ^15^N, ^13^C labeled MalE-Ct sample, with or without a 5.6-fold excess of dp14 (degree of polymerization), with standard Bruker sequences. Acquisition parameters for the HNCACB experiment were, for ^1^H (t3), 3 k points for 14 ppm, for ^15^N (t2) 106 points for 24 ppm, and for ^13^C (t1), 256 points for 70 ppm. For the HNN experiment, the t1 dimension is ^15^N, and 256 points were acquired for 24 ppm. Spectra were processed using Bruker TOPSPIN 2.1 software, after zero-filling to 4 k×512×512 points and multiplication with a squared sine bell window function.

### Determination of dissociation constants using NMR titration assays

For the titration experiments, 60 µM free ^15^N-labeled MalE-Ct was added to appropriate amounts of lyophilized dp14 to obtain dp14: MalE-Ct molecular ratios of 0.5, 1, 2, 5, and 10. ^1^H-^15^N HSQC spectra were acquired at 900 MHz and 20°C. The same methodology was followed with the ^15^N-labeled samples of *M. tuberculosis* and *M. avium* HBHA, but the spectra were acquired at 600 MHz.

### Data analysis

The binding constant was calculated by fitting the formula 
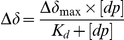
 to the observed resonances, with Δδ as the combined ^1^H_N_ and ^15^N chemical shift 
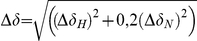


## Supporting Information

Table S1 Chemical shift predictions of the HBD, according to the random coil chemical shifts defined from a set of intrinsically unstructured proteins [42].
(DOC)

Figure S1 (A) The HNN plane extracted at the ^15^N frequency of the (K)A(P) resonance (127.04 ppm; green) defines the ^15^N frequency of the K(AP) resonance. Similarly, the plane extracted at the latter frequency (123.03 ppm; blue) connects with the (K)A(P) resonance, and defines the ^1^H frequency of the K(AP) residue (8.368 ppm). (B) The HNN plane extracted at the ^15^N frequency of the intense (K)A(AP) resonance (126.5 ppm; green) defines the ^15^N frequency of the two (KA)A(P) resonance. Similarly, the plane extracted at the third (K)A(AP) frequency (126.15 ppm; blue) connects with its (KA)A(P) resonance. The experiment equally connects the upstream K residues, degenerate for repeats 1 and 2 (at 123.9 ppm) and at 123.6 ppm for repeat 3.
(PDF)

Figure S2 (A) ^1^H, ^15^N HSQC spectra of the MalE-Ct (black) of the synthetic peptide at natural abundance (light blue) and of the synthetic peptide incorporating a single ^15^N-Ala per repeat (dark blue). Individual ^15^N-ala residues are indicated in blue on the primary sequence. (B and C) Selected panels of the ^1^H, ^15^N HSQC spectra of the MalE-Ct with an excess of dp14 (red) and of the synthetic peptide incorporating a single ^15^N-Ala per repeat with the same excess of dp14 (dark blue). (B) The (KK)A(AP) in the third repeat is ^15^N labeled, and defines together with the HNN this repeat. (C) Similarly, the (P)A resonance of the 2^nd^ repeat is uniquely labeled, and defines the second repeat in the MalE-Ct.
(PDF)

Figure S3Representative ^15^N HSQC NMR spectra for Ala-26 of the MalE-Ct HBD domain (60 µM) (A, B and C) with increasing amounts of derived oligosaccadrides shifting peaks used for the determination of the dissociation constant: dp10 (A), dp14 (B) at the following concentrations, free protein (black), 30 µM (red), 60 µM (blue), 120 µM(purple), 300 µM (green) and 600 µM (marrow). (C) free protein (black), equimolar ratio between MalE-Ct and dp4 (red), equimolar ration between MalE-Ct and dp10 (blue) and equimolar ratio between MalE-Ct and dp14 (green). Chemical shift differences were calculated as 
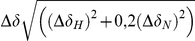
, where Δδ_H_ and Δδ_N_ are the observed chemical shift changes for ^1^H and ^15^N, respectively. For the determination of the dissociation constants (D), Δδ was plotted as a function of the molar ratio (dp∶protein) (E) Ala-6 (▪) and Thr-37 (▾) from the MalE-Ct HBD domain bound to dp14; (F) Ala-26 from the MalE-Ct HBD bound to dp14 (•) and dp10 (▴).(PDF)
