# Supplementary material for: Differential Contribution of the Repeats to Heparin Binding of HBHA, a Major Adhesin of Mycobacterium tuberculosis
Source: PLoS One. 2012 Mar 5;7(3):e32421. doi: 10.1371/journal.pone.0032421 (PMC3293801; doi:10.1371/journal.pone.0032421)
Supplement: Table S1 — Chemical shift predictions of the HBD, according to the random coil chemical shifts defined from a set of intrinsically unstructured proteins [42]. (DOC) [file pone.0032421.s001.doc]

Chemical shift predictions according to Tamiola K, Acar B, Mulder FA (2010) Sequence-specific random coil chemical shifts of intrinsically disordered proteins. J Am Chem Soc 132: 18000-18003.

2 K 176.29 32.84 56.42 122.66 8.34 4.16

3 A 177.49 19.02 52.61 125.21 8.28 4.15

4 A 175.61 18.54 50.34 124.34 8.22 4.45

5 P 176.79 31.96 63.15 135.97 -- 4.25

6 A 177.86 18.98 52.48 124.54 8.40 4.18

7 K 176.77 32.82 56.43 120.78 8.26 4.18

8 K 176.29 32.84 56.42 122.66 8.34 4.16

9 A 177.49 19.02 52.61 125.21 8.28 4.15

10 A 175.61 18.54 50.34 124.34 8.22 4.45

11 P 176.79 31.96 63.15 135.97 -- 4.25

12 A 177.86 18.98 52.48 124.54 8.40 4.18

13 K 176.77 32.82 56.43 120.78 8.26 4.18

14 K 176.29 32.84 56.42 122.66 8.34 4.16

15 A 177.49 19.02 52.61 125.21 8.28 4.15

16 A 175.61 18.54 50.34 124.34 8.22 4.45

17 P 176.79 31.96 63.15 135.97 -- 4.25

18 A 177.86 18.98 52.48 124.54 8.40 4.18

19 K 176.77 32.82 56.43 120.78 8.26 4.18

20 K 176.29 32.84 56.42 122.66 8.34 4.16

21 A 177.49 19.02 52.61 125.21 8.28 4.15

22 A 177.67 18.98 52.57 123.26 8.19 4.14

23 A 177.97 19.00 52.62 123.34 8.19 4.17

24 K 176.77 32.82 56.43 120.78 8.26 4.18

25 K 176.29 32.84 56.42 122.66 8.34 4.16

26 A 175.43 18.57 50.37 126.29 8.30 4.47

27 P 176.79 31.96 63.15 135.97 -- 4.25

28 A 177.86 18.98 52.48 124.54 8.40 4.18

29 K 176.77 32.82 56.43 120.78 8.26 4.18

30 K 176.29 32.84 56.42 122.66 8.34 4.16

31 A 177.49 19.02 52.61 125.21 8.28 4.15

32 A 177.67 18.98 52.57 123.26 8.19 4.14

33 A 177.97 19.00 52.62 123.34 8.19 4.17

34 K 176.77 32.82 56.43 120.78 8.26 4.18

35 K 176.44 32.86 56.39 122.62 8.34 4.23

36 V 176.33 32.66 62.42 121.79 8.25 4.04

37 T 174.37 69.65 61.90 118.44 8.29 4.21

38 Q 175.86 29.47 55.84 122.97 8.42 4.24
