# Supplementary figures and images for: Differential Contribution of the Repeats to Heparin Binding of HBHA, a Major Adhesin of Mycobacterium tuberculosis
Source: PLoS One. 2012 Mar 5;7(3):e32421. doi: 10.1371/journal.pone.0032421 (PMC3293801; doi:10.1371/journal.pone.0032421)

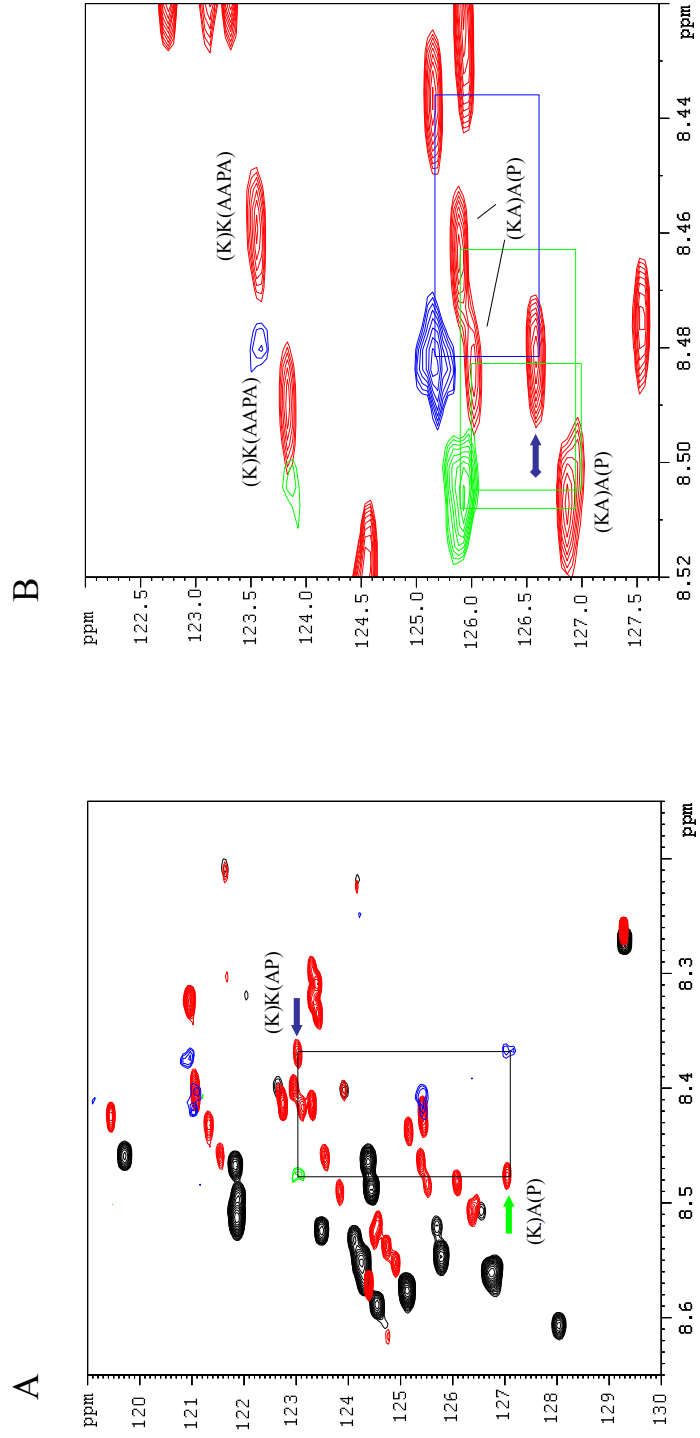

Figure S2

Supplement: Figure S1 — (A) The HNN plane extracted at the 15N frequency of the (K)A(P) resonance (127.04 ppm; green) defines the 15N frequency of the K(AP) resonance. Similarly, the plane extracted at the latter frequency (123.03 ppm; blue) connects with the (K)A(P) resonance, and defines the 1H frequency of the K(AP) residue (8.368 ppm). (B) The HNN plane extracted at the 15N frequency of the intense (K)A(AP) resonance (126.5 ppm; green) defines the 15N frequency of the two (KA)A(P) resonance. Similarly, the plane extracted at the third (K)A(AP) frequency (126.15 ppm; blue) connects with its (KA)A(P) resonance. The experiment equally connects the upstream K residues, degenerate for repeats 1 and 2 (at 123.9 ppm) and at 123.6 ppm for repeat 3. (PDF) [file pone.0032421.s002.pdf]

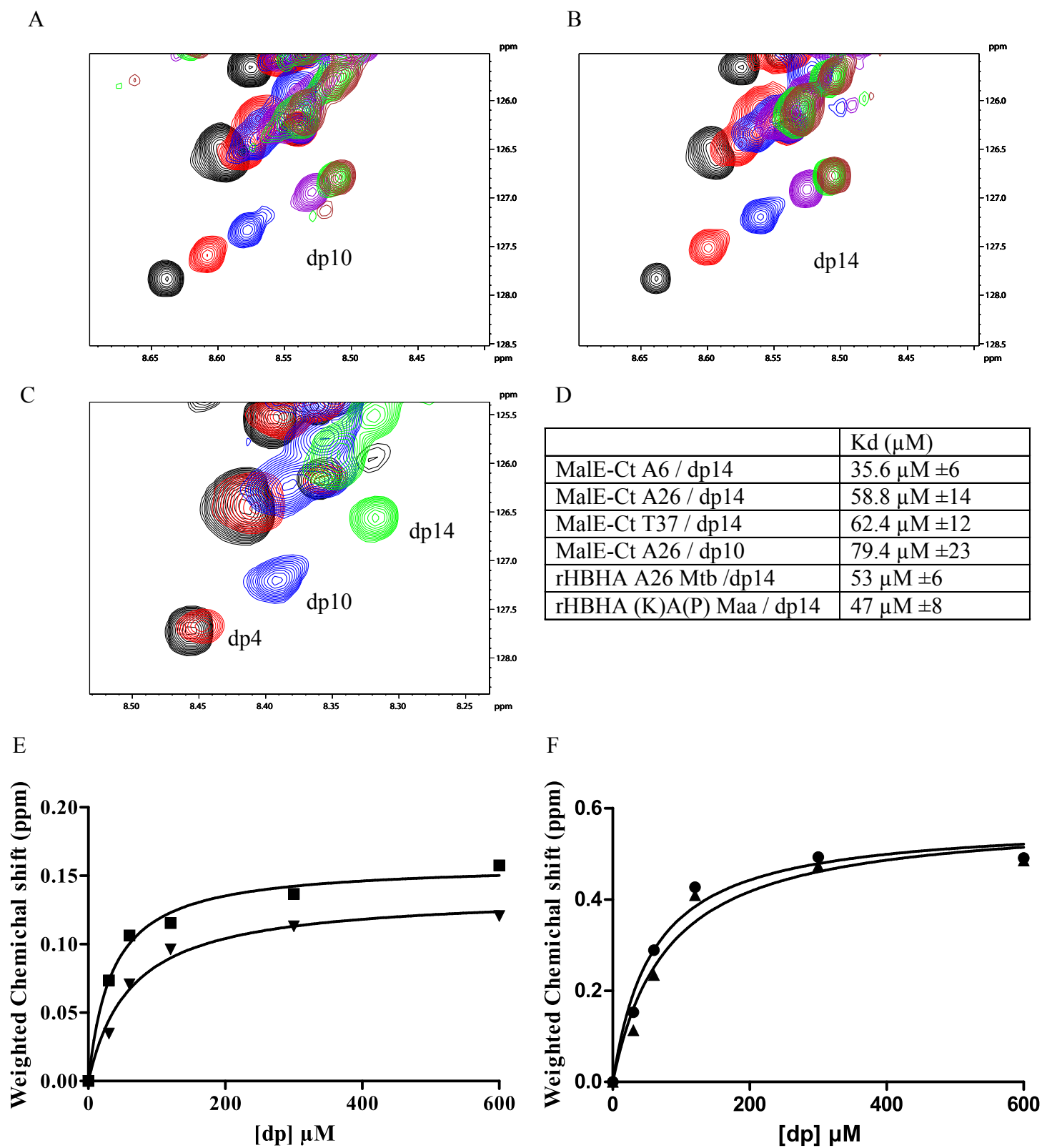

Figure S4

Supplement: Figure S3 — Representative 15N HSQC NMR spectra for Ala-26 of the MalE-Ct HBD domain (60 µM) (A, B and C) with increasing amounts of derived oligosaccadrides shifting peaks used for the determination of the dissociation constant: dp10 (A), dp14 (B) at the following concentrations, free protein (black), 30 µM (red), 60 µM (blue), 120 µM(purple), 300 µM (green) and 600 µM (marrow). (C) free protein (black), equimolar ratio between MalE-Ct and dp4 (red), equimolar ration between MalE-Ct and dp10 (blue) and equimolar ratio between MalE-Ct and dp14 (green). Chemical shift differences were calculated as , where ΔδH and ΔδN are the observed chemical shift changes for 1H and 15N, respectively. For the determination of the dissociation constants (D), Δδ was plotted as a function of the molar ratio (dp∶protein) (E) Ala-6 (▪) and Thr-37 (▾) from the MalE-Ct HBD domain bound to dp14; (F) Ala-26 from the MalE-Ct HBD bound to dp14 (•) and dp10 (▴). (PDF) [file pone.0032421.s004.pdf]
