# Supplementary material for: Differential Contribution of the Repeats to Heparin Binding of HBHA, a Major Adhesin of Mycobacterium tuberculosis
Source: PLoS One. 2012 Mar 5;7(3):e32421. doi: 10.1371/journal.pone.0032421 (PMC3293801; doi:10.1371/journal.pone.0032421)
Supplement: Figure S2 — (A) 1H, 15N HSQC spectra of the MalE-Ct (black) of the synthetic peptide at natural abundance (light blue) and of the synthetic peptide incorporating a single 15N-Ala per repeat (dark blue). Individual 15N-ala residues are indicated in blue on the primary sequence. (B and C) Selected panels of the 1H, 15N HSQC spectra of the MalE-Ct with an excess of dp14 (red) and of the synthetic peptide incorporating a single 15N-Ala per repeat with the same excess of dp14 (dark blue). (B) The (KK)A(AP) in the third repeat is 15N labeled, and defines together with the HNN this repeat. (C) Similarly, the (P)A resonance of the 2nd repeat is uniquely labeled, and defines the second repeat in the MalE-Ct. (PDF) [file pone.0032421.s003.pdf]

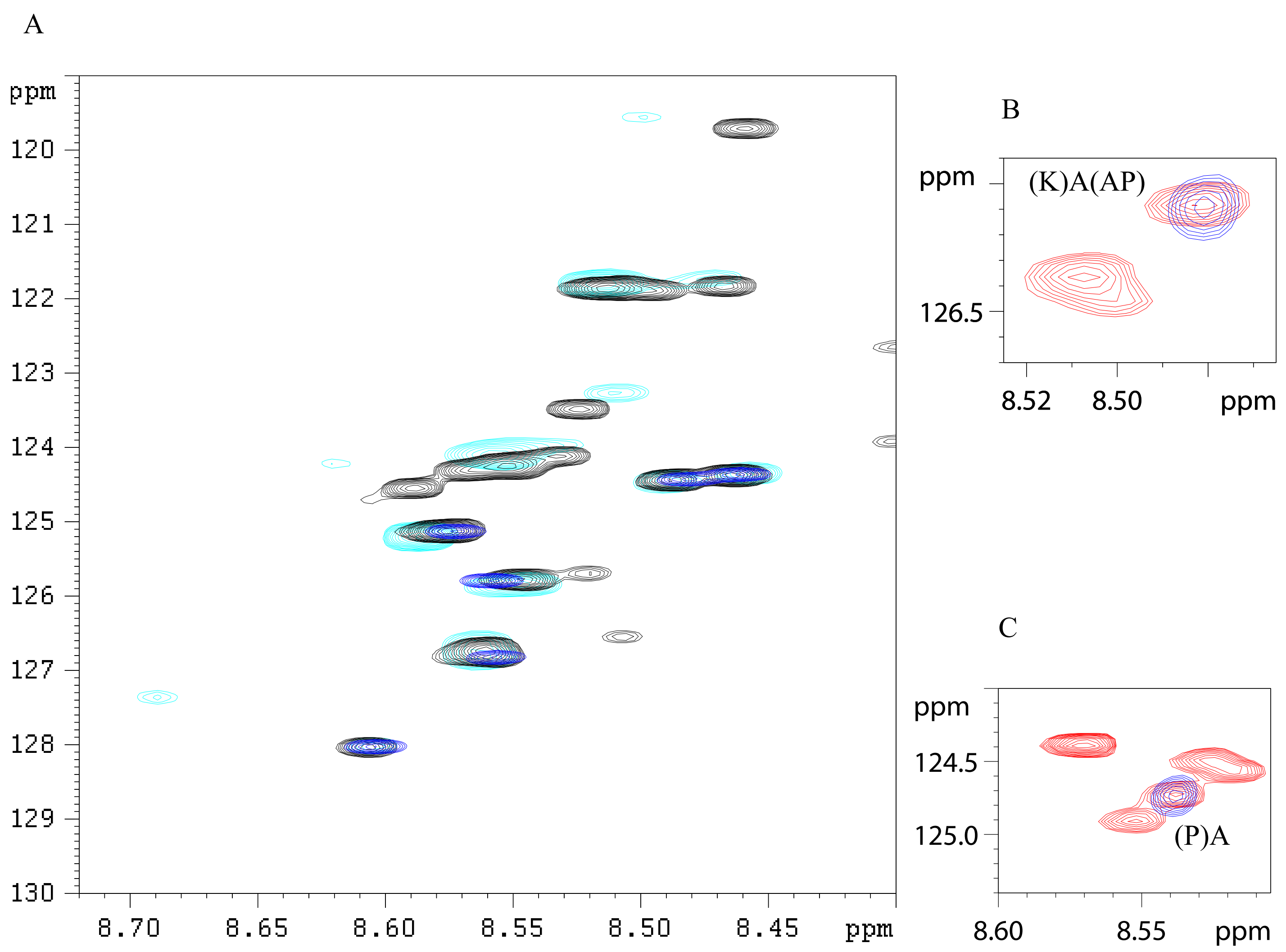

1-K KAAPAK KAAPAK KAAPAK KAAAK KAPAK KAAAK KVTQK-39

Figure S3
